# Molecular Functions and Pathways of Plastidial Starch Phosphorylase (PHO1) in Starch Metabolism: Current and Future Perspectives

**DOI:** 10.3390/ijms221910450

**Published:** 2021-09-28

**Authors:** Noman Shoaib, Lun Liu, Asif Ali, Nishbah Mughal, Guowu Yu, Yubi Huang

**Affiliations:** 1 College of Agronomy, Sichuan Agricultural University, Chengdu 611130, China; nomanshoaib@stu.sicau.edu.cn (N.S.); ll2950385@163.com (L.L.); nishbahmughal97@gmail.com (N.M.); 2 State Key Laboratory of Crop Gene Exploration and Utilization in Southwest China, Rice Research Institute, Sichuan Agricultural University, Chengdu 611130, China; asifali@sicau.edu.cn; 3 State Key Laboratory of Crop Gene Exploration and Utilization in Southwest China, Sichuan Agricultural University, Chengdu 611130, China

**Keywords:** starch phosphorylase, plastidial starch phosphorylase, amyloplastic phosphorylase, synthetic activity, phosphorolytic activity

## Abstract

Starch phosphorylase is a member of the GT35-glycogen-phosphorylase superfamily. Glycogen phosphorylases have been researched in animals thoroughly when compared to plants. Genetic evidence signifies the integral role of plastidial starch phosphorylase (PHO1) in starch biosynthesis in model plants. The counterpart of PHO1 is PHO2, which specifically resides in cytosol and is reported to lack L80 peptide in the middle region of proteins as seen in animal and maltodextrin forms of phosphorylases. The function of this extra peptide varies among species and ranges from the substrate of proteasomes to modulate the degradation of PHO1 in *Solanum tuberosum* to a non-significant effect on biochemical activity in *Oryza sativa* and *Hordeum vulgare*. Various regulatory functions, e.g., phosphorylation, protein–protein interactions, and redox modulation, have been reported to affect the starch phosphorylase functions in higher plants. This review outlines the current findings on the regulation of starch phosphorylase genes and proteins with their possible role in the starch biosynthesis pathway. We highlight the gaps in present studies and elaborate on the molecular mechanisms of phosphorylase in starch metabolism. Moreover, we explore the possible role of PHO1 in crop improvement.

## 1. Introduction

Phosphorylase (EC 2.4.1.1), designated as starch phosphorylase (PHO) in plants and glycogen phosphorylase (gPHO) in animals, has been studied for decades due to its multiple functions. Phosphorylase was first discovered in the liver tissue of mammals in 1939 and was reported to work in the reversible transfer of the glucosyl unit from glucose-1-phosphate to glycogen with the release of inorganic phosphate [1]. In the 1940s, the same type of reaction was described in higher plants [2,3]. Phosphorylase shares a solid evolutionary relationship among animals, plants, algae, and bacteria. In comparison to animals, higher plants and green algae have two distinct forms of starch phosphorylases, a plastidial or amyloplastic form (PHO1 or SP-L; low affinity for glycogen) and a cytosolic form (PHO2 or SP-H; high affinity for glycogen) [4]. These two forms have significant sequence similarities to each other but largely differ in their molecular sizes, substrate specificities, and physiological roles [5]. The alignment of proposed phosphorylase sequences from a wide range of species have shown that phosphorylase in higher plants is structurally similar to the animals and bacterial forms (maltodextrin phosphorylase) but differ in transit peptides of up to 50 amino acids in the N-terminus [6,7,8] and L80 domain (an extra peptide of up to 82 amino acid residues). The L80 domain is present in the middle of PHO1 and absent in animal, bacteria, and PHO2 forms of higher plant phosphorylases [9]. The presence of this prominent extra peptide in the middle region of PHO1 can be responsible for the diverse biochemical activities of PHO1 [10,11]. Previous findings have claimed that L80 peptide contains a variable set of negatively charged amino acids, phosphorylation sites, and a potential PEST motif (rich in proline, glutamic acid, serine, and threonine) [12]. It has been reported that the L80 insertion sterically hinders the binding of large polysaccharides with PHO1 in *Solanum tuberosum* [13]. In addition, L80 insertion was found to be an active substrate for proteasomes (20S or 26S), when one of the N-terminal amino acid residues had been modified by phosphorylation in *Ipomoea batatas* [14]. The PHO1 in *Solanum tuberosum* maintains its intact structure in young tubers but steadily degrades into smaller peptides as the tubers become mature [7]. Both the intact (105 kDa) and degraded (55 kDa) forms of PHO1 have been reported in *Vigna radiata*. Interestingly, both forms were identified to make active catalytic complexes [15]. In contrast, the PHO1 of cereals including *Oryza sativa* [16,17], *Zea mays* [8], *Hordeum vulgare* [18], and *Triticum aestivum* [9] is not degraded by the proteasome, even though they all have a PEST motif in L80 insertion. A detailed study would be required to uncover these differences to reach a conclusion.

PHO1 accounts for 96% of total phosphorylase activity in *Oryza sativa* and is restricted to amyloplast stroma [16]. The PHO1 in *Zea mays* is the second most abundant protein in amyloplast stroma after SBEIIb (an isoform of the subgroup of starch branching enzyme (SBEII)) [8]. Moreover, the availability of PHO1 correlates with the expression levels of other starch biosynthetic enzymes in higher plants [19,20,21]. These findings make PHO1 a major active form of phosphorylase. In this review, we made efforts to information about phosphorylase, especially PHO1. In this regard, we reviewed the data of the genes, proteins, their structures, the role of phosphorylation, protein–protein interaction, and redox modulation in the starch biosynthesis pathway. Our review can be utilized in molecular breeding to obtain an optimized starch yield. Furthermore, it will help to establish an understanding of the primary functions, binding partners, and molecular basis of the biosynthesis pathway regulated by PHO1.

## 2. Starch Metabolism and Phosphorylase

### 2.1. Starch Biosynthesis

Starch is an important component of plants and has been subjected to profound research; however, understanding its granule synthesis remains obscure. Amylose and amylopectin are the major components of starch [22]. Some minor components such as minerals, lipids, and proteins are also important parts of starch granules (comprising a small percentage of the total weight of the granule) [23]. Starch biosynthesis is a complex mechanism that involves the activity of more than 15 enzymes [24]. These enzymes, each with various tissue-specific and developmental specific isoforms of starch synthases (SSs, EC 2.4.1.21), starch branching enzymes (SBEs, EC 2.4.1.18), and starch debranching enzymes (DBEs, 3.2.1.41; mainly isoamylases (ISAs)) are the major groups of enzymes involved in starch metabolism. ADP-Glc pyrophosphorylase (AGPase, EC 2.7.7.27) is the key enzyme in the first mandatory step of starch biosynthesis that produces the adenosine diphosphate glucose ADP-Glc from glucose-1-phosphate (Glc-1-P) and ATP [25]. ADP-Glc acts as a substrate to extend the α-1,4 linked glucans through SSs. There are five main isoforms of SSs essentially present in all crops, namely SSI, SSII, SSIII, SSIV, and granule bound starch synthase (GBSS). Among these SSI, SSII, and SSIII are involved in the synthesis of amylopectin [26,27], whereas GBSS is involved in amylose synthesis as the mutants that show loss of function of GBSS produce amylose-free starch [28,29]. SBEs introduce the branch point and cleave the elongated α-1,4 linked glucans and transfer that segment to the sides of an acceptor chain through α-1,6 linkage. DBEs further cleave the α-1,6 linked chains that are needed for proper amylopectin structure. In addition, they are thought to promote the crystallization of amylopectin through trimming and degrading the branched glucan [30,31]. Two of three isoforms of ISA, ISAI and ISAII, take part in amylopectin synthesis, whereas ISAIII is involved in the degradation of starch [32]. Furthermore, a study on the expression level of PHO1 in *Hordeum vulgare* [33,34], *Arabidopsis thaliana* [35], *Triticum aestivum* [9], and *Oryza sativa* [16] has indicated the involvement of PHO1 in starch metabolism. The comprehensive details of the structure and synthesis of starch can be found in recent reviews [31,36].

### 2.2. Initiation of Starch Granule in Comparison to Glycogen

Based on biochemical evidence, it was assumed that phosphorylase was involved in the initiation and amplification of glycogen and starch biosynthesis in animals and plants, respectively [9,16,37,38]. However, the discovery of glucosyl-transferase in the liver tissue ruled out this assumption for the glycogen synthesis of gPHO. Indeed, glycogenin is a precise initiator protein in the de novo biosynthesis of glycogen in fungi and animals in a process of self-glucosylation with UDP-glucose to generate covalently bound malto-oligosaccharides (MOs) [39]. Bacteria likely utilize the glycogen synthase (GS) to transfer the sugar moiety from ADP-Glc to distinctive binding amino acid residues in the process of de novo MO synthesis [40]. There are no experimental reports available to date that indicate the participation of phosphorylase in the glycogen initiation process. On the contrary, no self glucosylation proteins have been reported in plants [41]. Therefore, unlike animals the starch initiation mechanism in the plants could be different from the glycogen initiation mechanism in bacteria, fungi, and animals. In addition, there are indications that AGPase is not entirely involved in starch biosynthesis as it requires a primer of reasonable chain length (G2–G4) [42]. However, a study on potato tuber phosphorylase claimed that de novo synthesis of amylose occurs in the absence of primer [43]. AGPase and SSs were not present in the early stages of development in maize kernel, but some quantity of starch was synthesized already [44]. Moreover, they further suggested that the synthesis of starch must follow a different route at the start [44].

**Figure 1:**
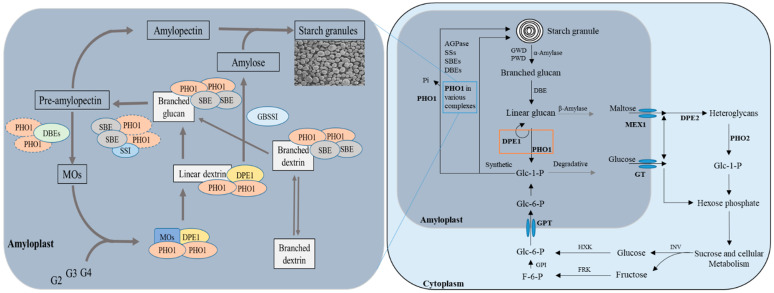
Putative roles of PHO1 and PHO2 in starch biosynthesis and degradation in the non-photosynthetic cell. Abbreviations: GWD: glucan water dikinase, PWD: phospho-glucan water dikinase, DBE: debranching enzyme, DPE1, plastidial disproportionating enzyme, PHO1, plastidial starch phosphorylase, AGPase: ADP-Glc pyrophosphorylase, SSs: starch synthases, SBEs: starch branching enzymes, DPE2: cytosolic disproportionating enzymes, PHO2: cytosolic starch phosphorylase, INV: invertase, HXK: hexose kinase, FRK: fructose kinase, GPI: glucose-6-phosphate isomerase, GT: glucose transporter, MEX1: maltose transporter, GPT: glucose-6-phosphate transporter, Pi: inorganic phosphate, Glc-1-P: glucose-1-phosphate, Glc-6-P: glucose-6-phoshphate, F-6-P: fructose-6-phosphate and MOs: malto oligosaccharide, G2: maltose, G3: maltotriose, and G4: maltotetraose. The orange box indicates the concerted action of DPE1 and PHO1, which might define the fate of the metabolites. The trimmed MOs that are generated by the action of DBEs and PHO1 and branched oligosaccharides enter the starch biosynthetic pathway together, where they are first elongated by the concerted action of DPE1 and PHO1 metabolon, followed by the further elongation of by the SBEs and PHO1 complex. Alternatively, branched oligosaccharides are further elongated and branched by the combined action of PHO1 and isozymes of SBEs. Fusion of the generated products leads to the formation of pre-amylopectin via the possible complex of PHO1, SSs, and SBEs isozymes (still unknown). Trimming of MOs on the pre-mature amylopectin leads to its maturation that further combines with amylose (generated via the action of GBSSI) to form the starch granules. Produced from [12,17,31,36,42,50,51,52].

There can be two possible routes for starch synthesis (Figure 1), either through the PHO1 or AGPase/SSs, which depend on the availability of substrate-level (Glc-1-P) [45,46,47]. The PHO1 route is more energy-efficient than AGPase/SSs as it required a higher concentration of Glc-1-P [12]. PHO1 could have a special importance during the early stages of endosperm development because the activity of phosphorylase is 40-fold greater than SSs in initial stages and 10-fold greater in lateral stages of development in developing maize kernels [48]. A mutant (*sta4*) lacking the PHO1 displayed reduced levels of starch with abnormally shaped granules with high amylose content, arguing for a role of PHO1 in starch biosynthesis in *Chlamydomonas* [49]. *Shrunken 4* (*sh4*), a mutant of *Zea mays* with no activity of phosphorylase resulted in the reduced starch accumulation and shrunken morphology of maize kernels [48], suggesting the PHO1 can determine the granule shape/morphology.

### 2.3. Multiple Complex Formation

Increasing evidence supports that starch biosynthesis does not consist of some simple and isolated reactions. The protein–protein interaction and regulatory coordination among starch biosynthetic enzymes have been reported as the key features in starch biosynthesis [5,14,53]. A genetic study has reflected that disproportionating enzyme 1 (DPE1, EC 2.4.1.25) and PHO1 are potentially involved in the initiation of storage starch biosynthesis in plants [17]. Based on previously reported work [10,50], it has been revealed that PHO1 forms a complex with DPE1 and serves as a metabolon in the recycling of MOs back to growing amylopectin through Glc-1-P synthesis in *Oryza sativa*. The phosphorolytic reaction of PHO1 was stimulated by DPE1 in Chlamydomonas [54]. It has also been shown that PHO1 forms complexes with various SBEs and SSs in a phosphorylation manner to regulate the starch biosynthesis and is involved in elongation and branching of MOs alternatively, followed by further elongation via functional interaction between PHO1 and isozymes of branching enzymes (Figure 1). Another proposed mechanism explains the direct action of PHO1 on the surface of starch to add glucose units from Glc-1-P with the release of inorganic phosphate (Pi) [9]. These observations suggest a certain role of PHO1 in starch biosynthesis and metabolism of MOs.

### 2.4. Starch Degradation

Several starch-degrading enzymes have been identified [55,56] and can be generally classified into two categories: phosphorolytic and hydrolytic degrading enzymes. The branched and linear glucans are produced from the starch degradation through the actions of newly identified candidates: glucan water dikinase (GWD; EC 2.7.9.4) and phosphoglucan water dikinase (PWD; EC 2.7.9.5) [57]. However, α-amylase (EC 3.2.1.1) is thought to be the first enzyme for starch degradation. Branched glucans are further reduced to linear glucans by DBEs. PHO1 and β-amylase produce the neutral sugars from linear glucans and eventually generate metabolites that are transported to the cytosol by their specific transporters [35]. Concerted action of cytosolic forms of PHO and DPE produce heteroglycans (heteropolysaccharides containing two or more different monosaccharide units) and hexose phosphates, which subsequently enter sucrose and cellular metabolism pathways [51]. To keep the balance between cytosol and amyloplast, cellular metabolites can enter the starch biosynthesis pathway through the actions of invertase (INV), hexose kinase (HXK), and fructose kinase (FRK) enzymes (Figure 1).

### 2.5. Additional Role of PHO1

Besides storage starch synthesis in non-photosynthetic cells, it is stated that PHO1 may have an additional role in modulating the photosynthetic activity by interacting with the PsaC region of Photosystem-I (PSI) in photosynthetic cells of *Oryza sativa* [12,36]. It could influence the PSI activity indirectly, via the pentose phosphate pathway (PPP), as the PHO1^−^ (lacking in PHO1) mutant (*bmf136*) of rice was reported to have different PSI activity and exhibited lower photosynthetic performance in comparison to wild type [16,45]. The phenotype of *bmf136* was rescued to normal when it was complemented with the PHO1. However, when these plants were complemented with the PHO1∆L80 (PHO1 with excised L80 region) instead of typical, enhanced photosynthetic activity was observed. These findings suggest that PHO1 also acts as a modulator of photosynthesis, and the L80 domain is a controller in the photosynthesis modulation.

## 3. Synthetic and Phosphorolytic Activity of Phosphorylase

Both PHO1 and PHO2 can catalyze the phosphorolysis of glucans to generate Glc-1-P and Pi in a reversible reaction. Since the reaction is reversible, the appropriate catalyzing direction under physiological conditions is still in debate [12]. In the synthetic direction, phosphorylase is involved in the transfer of the glucosyl unit from Glc-1-P to the growing glucan chain with the release of inorganic phosphate [49,58,59]. In the phosphorolytic direction, the same reaction takes place in a reverse direction. Which reaction is favored in starch biosynthesis is dependent on the Pi/Glc-1-P ratios; a higher pi/Glc-1-P ratio favors the reaction in the phosphorolytic direction and a low Pi/Glc-1-P ratio favors the reaction in the synthetic direction [60]. However, evidence suggests that the direction of PHO1 activity can be also controlled by some other factors than available substrate levels [36]. In comparison to PHO1, PHO2 of higher plants has been reported to degrade the maltose phosphorolytically, a result of starch degradation [37,61]. However, PHO1 is supposed to play an efficient synthetic role in starch biosynthesis because of its expression and localization, in parallel to storage starch synthesis and accumulation therein [9]. A study of PHO1 in *Zea mays* has shown that it stimulates the reaction in the phosphorolytic direction with MOs [5,62], and in *Oryza sativa* PHO1 showed a preferable role in the synthetic direction over degradation even with the high level of Pi [41,47]. Phosphorylase from *Tetraselmis subcordiformis* (marine green microalga) preferred a two-fold higher affinity in the synthetic direction over phosphorolytic direction [63]. Further to supporting the synthetic role, PHO1 has been seen to be involved in actively taking up the Glc-1-P in tuber parenchyma cells and depositing it to storage starch granules in *Solanum tuberosum* [64]. PHO1 actively interacts with other starch synthetic enzymes to form the functionally active protein–protein complex and to modulate its synthetic activity [17,50,65,66]. PHO2 precisely exists in the cytoplasm where it can interact with DPE2 and modulates the degradation reactions only [17,36].

Further, to elaborate the synthetic and phosphorolytic activity of phosphorylase [67], Subsinghe, R.M., performed experiments on *Zea mays* PHO1 at various developmental stages and reported that PHO1 remains active throughout the endosperm development and showed synthetic activity. At 22 days after anthesis (DAA), PHO1 showed both synthetic and phosphorolytic activity when tested on native affinity zymograms [18]. In *Hordeum vulgare*, PHO1 also confirmed the synthetic activity in developing endosperm, which was highest at 12 DAA and gradually decreased afterward, but the activity does not correlate with the expression level, possibly because of substrate preferences. The preference of substrate by PHO1 has been intensively investigated in various plant species [8,49,68,69,70,71]. PHO1 in *Ipomoea batatas* showed low binding affinity toward starch (high molecular weight) and high binding affinity towards MOs (low molecular weight) [68]. In contrast, PHO1 in *Zea mays* showed high binding affinity toward high molecular weight compounds and low binding affinity toward low molecular weight compounds [69]. In *Zea mays* [8], *Solanum tuberosum* [70], and *Spinacia oleracea* [71] synthetic activity of PHO1 was reported to be high when amylopectin was used as a substrate in comparison to highly branched glycogen. In *Solanum tuberosum* L80 insertion was reported to block the binding affinity of PHO1 with high molecular weight compounds [68]. In cereals, whether L80 insertion affects the substrate affinity has not been identified or described yet.

## 4. Genomics of PHO1

During the past few years, great efforts have been made to sequence the whole genomes of plants. The availability of data is important in answering the biological questions in comparative studies [72,73]. In parallel, scientists have discovered the gene encoding phosphorylase (*PHO*) and interpreted the gene structure, identity, localization, conserved regions, and the expression in wide range of species including maize (*Zea mays*; NP_001296783.1) [8,58,74], brome (*Brachypodium distachyon*; XP_003559211.1) [75], barley (*Hordeum vulgare*; KAE8783983.1) [18], rice (*Oryza sativa*; XP_015631420.1) [16,47,52], wheat (*Triticum aestivum*; ACC59201.1) [9], potato (*Solanum tuberosum*; NP_001275215.1) [7,76,77], sweet potato (*Ipomoea batatas*; M64362.1) [14,78], orange (*Citrus clementina*; NW_0062622139.1), mung bean (*Vigna radiata*; LOC106770459) [15,79], cotton (*Gossypium Arboreum*; LOC108456840) [80], bunch grass (*Panicum hallii*; LOC112876003), tomato (*Solanum lycopersicum*; XP_0042234848.1) [81], and wild tomato (*Solanum habrochaites*; MG962532.1) [81]. g*PHO* in animals has been investigated comprehensively as compared to *PHO1* in plants. Only *PHO1* of *Zea mays*, *Oryza sativa*, and *Hordeum vulgare* has been investigated comprehensively in main cereal crops. *PHO1* for *Triticum aestivum*, *Setaria italic*, and *Ipomoea batatas* has not been demonstrated and characterized yet and should be the target of future research.

### Characteristics of PHO1

**Figure 2:**
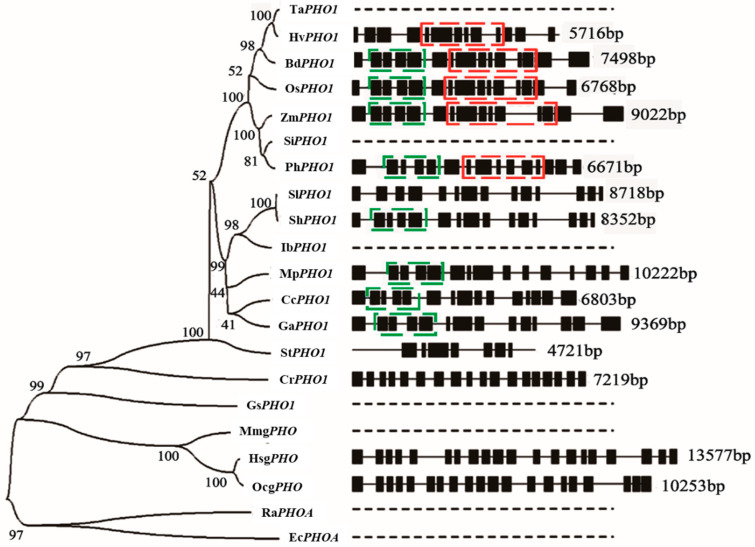
Phylogram showing the evolutionary pattern of *PHO1*, g*PHO*, and maltodextrin forms of phosphorylases and gene structures with common exon–intron junctions among a wide range of species. The tree was generated by MEGA 7 with the neighbor-joining method [83]. The bootstrap value is calculated based on 1000 repetitions and displayed on each node. Abbreviations: Ta: *Triticum aestivum*, Hv: *Hordeum vulgare*, Bd: *Brachypodium distachyon*, Os: *Oryza sativa*, Zm: *Zea mays*, Si: *Setaria italica*, Ph: *Panicum halli*, Sl: *Solanum lycopersicum*, Sh: *Solanum habrochaites*, Ib: *Ipomoea batatas*, Mp: *Mucuna pruriens*, Cc: *Citrus clementina*, Ga: *Gossypium arboreum*, St: *Solanum tuberosum*, Cr: *Chlamydomonas reinhardtii*, Gs: *Galdieria sulphuraria*, Mm: *Mus musculus*, Hs: *Homo sapiens*, Oc: *Oryctolagus cuniculus*, Ra: *Rhizobium anhuiense*, and Ec: *Escherichia coli*. Gene annotations with varying bp (base pairs). Green and red boxes are showing the common intron–exon junctions with identical intron phases (presenting highly conserved regions among phosphorylases in range of species) in the given gene structures. Dashed lines indicate the limited or uncharacterized gene structures.

Multiple sequence alignment has shown the variations in the total numbers of base pairs among *PHO1* in a wide range of species (Figure 2). Gene structure analysis among aligned species has revealed that *PHO1* of *Zea mays*, *Oryza sativa*, *Brachypodium distachyon*, *Citrus clementina*, and *Gossypium Arboreum* shared 15 and 14 number of exons and introns, respectively. Interestingly, *Hordeum vulgare* has 13 exons and 12 introns [33]. *PHO1* in *Solanum tuberosum* has shown seven exons and nine introns. In addition, alignment has revealed that some regions (intron or exon) of *PHO1* are highly conserved among species. It can clearly be seen that two exon–intron junctions are very much conserved among species. One is from exon–intron-2 to exon–intron-4, and the second is from exon–intron-6 to exon–intron-12. Alignments of exon–intron junctions among *PHO1* of *Oryza sativa*, *Zea mays*, *Hordeum vulgare*, *Solanum tuberosum*, *Arabidopsis thaliana*, and *Brachypodium distachyon* have revealed an identity of 56% (exon 2) to 90% (exon 12) with an average of 55%, while their intron showed the identity of 29% to 62.1% with an average of 48%. Multiple motifs have also been predicted in the 2 kb upstream region of the transcription start site in *PHO1* [33]. Proposed motifs (identified as described by [33]) suggest the regulating pattern of the *PHO1*, which can be influenced by abscisic acid (ABA), salt, and drought conditions (Table 1).

Microarray-based data have indicated the uniform pattern of *PHO1* transcripts in various tissues and seed developmental stages among *Hordeum vulgare* (GSE10328), *Oryza sativa* (GSE6901), and *Arabidopsis thaliana* (GSE9646). All studied *PHO1* and *PHO2* are proposed to have the regions (in promotors) that have functions in ABA-induced expression, but a study on *PHO1* of *Hordeum vulgare* suggested no effect of ABA on the expression of *PHO1* but up-regulation of *PHO2* [33]. However, in *Arabidopsis thaliana* significant down-regulation for both isoforms were detected. In contrast, the ABA can up-regulate the expression of both the *PHO1* and *PHO2* in *Zea mays* [82]. The effect of abiotic stresses on the expression of *PHO* has not been determined for various crops and could be worthy of investigation.

**Table 1:** Proposed motifs in 2 kb upstream region of transcription start site in *PHO1* of *Zea mays*, *Oryza sativa*, *Hordeum vulgare*, *Triticum aestivum*, and *Arabidopsis thaliana*.

Motif	Sequence	Possible Function and DB ID
aBrelaterD1	ACGTG	ABA, drought, and dark-induced regulation (S000414)
BIHD1OS	TGTCA	Disease resistance (S000498)
MYcatrD22	CACATG	ABA and drought regulation (S000174)
SOrlIP1at	GCCAC	Specific guard cell expression (S000483)
taaagStKSt1	TAAAG	Specific guard cell expression (S000387)
tataBOX5	TTATTT	Light response (S000112)
WBOXHVISO1	TTGAC	Environmental stress response (S000442)
WrKY71OS	TGAC	ABA regulation (S000447)
acgtaterD1	ACGT	Drought response (S000415)
cUrecOrecr	GTAC	Copper- and oxygen-induced coordinated response (S000493)
DOFcOreZM	AAAG	Carbon metabolism (S000265)
gataBOX	GATA	Light response and tissue-specific expression (S000039)
gt1gMScaM4	GAAAAA	Pathogen or salt-induced expression (S000453)
gtgantg10	GTGA	Pollen-specific regulation enhancement (S000378)
nODcOn2gM	CTCTT	Specific nodule expression (S000462)
OSe2rOOtnODUle	CTCTT	Organ-specific expression (S000468)
POllen1lelat52	AGAAA	Response against pollen (S000245)
raV1aat	CAACA	Binding to specific bipartite sequence (S000314)
rOOtMOtIFtaPOX1	ATATT	Organ-specific expression (S000098)
caatBOX1	CAAT	Tissue-specific promoter (S000028)

Note: All proposed motifs are identified as described by [33]. The only motif sequences that were repeated more than five times are mentioned here. DB IDs are the accession numbers from the PLACE database (https://www.dna.affrc.go.jp/PLACE/?action=newplace accessed on 15 June 2021 [84]).

## 5. Structure and Comparison of PHO1

**Figure 3:**
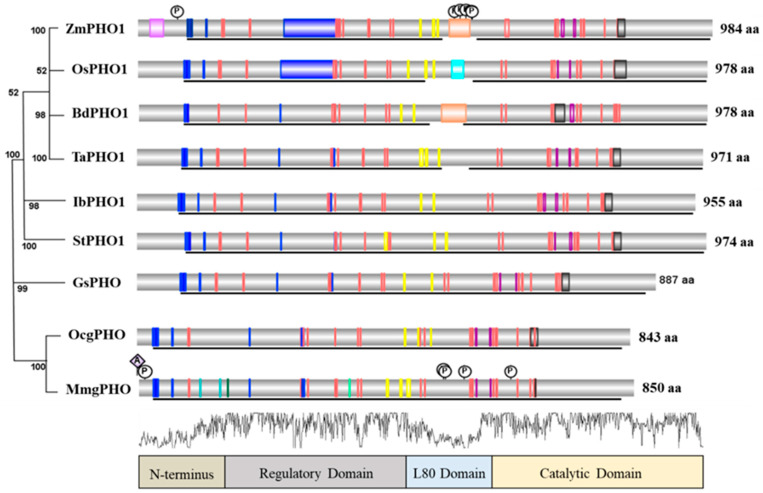
Annotated protein structure of phosphorylases from a wide range of species. Arranged in phylogenetic order. Representation is divided into four basic domains. Abbreviations: Zm: *Zea mays*, Os: *Oryza sativa*, Bd: *Brachypodium distachyon*, Ta: *Triticum aestivum*, Ib: *Ipomoea batatas*, St: *Solanum tuberosum*, Gs: *Galdieria sulphuraria*, Oc: *Oryctolagus cuniculus*, and Mm: *Mus musculus*. Amino acid numbers are varying among species. Vertical colored bars represent the different functional sites. Blue bars represent the homodimer interface for peptide binding, orange bars represent the catalytic regions, sky blue for coil entry, green for allosteric control region, gray for modification sites with pyridoxal phosphate (PLP), pink for glycine-rich domain/glutamic-acid-rich sites, yellow for glycogen storage sites, and purple for purine nucleotide inhibitor sites for caffeine. P represents the phosphorylation sites and A the acetylation sites. Brown brackets represent the PEST motif. Black lines indicate the highly conserved region that belongs to the GT35-glycogen-phosphorylase superfamily. Overall N-terminus and L80 domains are highly variable. Regulatory and catalytic domains display a high similarity index (more than 88%).

**Figure 4:**
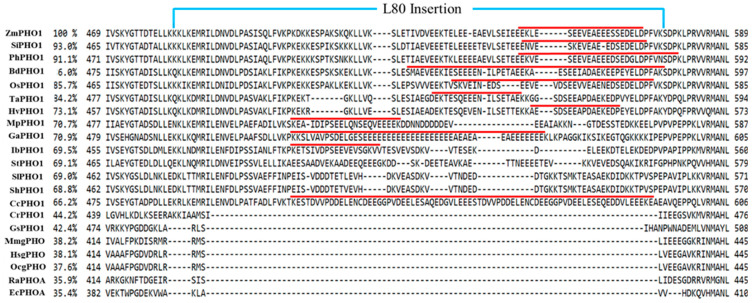
Alignment of middle regions (L80 peptide) of PHO1 from a wide range of species. Abbreviations are the same as in Figure 2. The percentage represents the identical index of the proteins that was calculated as a reference to *Zea mays* PHO1. Numbers express a portion of the amino acid sequence in the primary structure of proteins. Red lines indicate the potential PEST motif predicted by the software (https://emboss.bioinformatics.nl/cgi-bin/emboss/epestfind) accessed on 15 June 2021.

The comparison of the PHO1 of *Zea mays* [85] with other plant, microorganism, and animal forms of phosphorylases indicated that they vary in size. Alignment of main phosphorylase protein sequences has revealed the conserved junctions for various functional domains or sites of the protein including the catalytic site, residue binding site, coil entry, glycine-rich peptides, glutamic acid-rich peptides, repeats, L80 peptides, pyridoxal phosphate (PLP) binding residues, acetylation, and phosphorylation sites (Figure 3). Four regions, the N-terminal, regulatory domain, L80 insertion, and catalytic domain, can be determined as indicated in the phosphorylase protein structure comparison analysis (Figure 3). It was found that PHO from each species consists of a highly conserved GT-35-glycogen-phosphorylase domain [86]. In addition, regulatory and catalytic domains have been found highly conserved among all proteins. The N-terminus and L80 insertion have been found to vary among species. Only the PHO1 of the plants has L80 insertion as compared to PHO2 of plants, maltodextrin phosphorylase, and gPHO (Figure 4). The PEST motif is found on the L80 insertion of PHO1 in various plants [12]. The PLP binding junction has been found conserved among all PHO proteins, but it tends to vary in residue numbers. The regulatory domain mainly consists of glycogen storage and glucose-6-phosphate (Glc-6-P) binding sites. However, the catalytic domain mainly consists of glucose binding (active site), PLP binding, and nucleoside inhibitor residues. Alignment of sequences and information for functional sites indicated that limited research on plant phosphorylases has been conducted as compared to the animal form of phosphorylase. In addition, for 3D structure insights of phosphorylase protein, mammalian phosphorylase has received considerable attention, and *Hordeum vulgare* is the only plant in which the 3D structure of PHO1 has been established completely [18].

In relation to evolution, a highly divergent phylogenetic pattern has been observed among the phosphorylases (Figure 2). PHO1 of *Zea mays* showed a closer relationship with the PHO1 in *Panicum hallii* and *Setaria italica*. *Hordeum vulgare* showed a closer relationship with the PHO1 of *Triticum aestivum* than those in *Zea mays* and *Oryza Sativa*. All of the mentioned phosphorylases are likely to be evolved from a single species.

## 6. Regulation of Starch Phosphorylase

### 6.1. Phosphorylation

In plants, modification through phosphorylation was first reported in the major starch biosynthetic enzyme by [46,66] when they investigated the phosphorylation-dependent regulation of starch biosynthesis in the wheat plant. It was reported that serine residue in isoforms of SBEs including SBEI, SBEIIa (a subdivision of SBEII with slightly differing catalytic properties), and SBEIIb (a subdivision of SBEII with slightly differing catalytic properties), was phosphorylated. Phosphorylation stimulates, whereas dephosphorylation reduces, the activity of SBEIIa [46]. Recently, the brittle-2 (Bt2) subunit of AGPase was found phosphorylated in *Zea* mays, suggesting that phosphorylation does stimulate the AGPase activity in the starch metabolism [25]. Similarly, phosphorylation of the PHO1 has also been reported as the key feature during the starch filling process [45,66]. Phosphorylation is either the key for protein activation or a regulator for another interacting partner to stimulate the activity. In animals, gPHO was reported to activate the glycogen synthase, and phosphorylation of gPHO was the switch to activate the glycogen synthase in glycogen synthesis and degradation [87,88]. A study on plant and animal phosphorylases has confirmed that PHO can exist in homodimer and homotetramer states [67]. Phosphorylation sites were reported to form a side chain confirmation in assisting the intra subunit interaction along with a homodimer of gPHO [89]. In higher plants, it was found that the L80 insertion in the middle of PHO1 is rich in serine and threonine residues. These residues can be possible sites for phosphorylation modifications. Three-dimensional modeling suggests the side chain conformation for the L80 domain in *Hordeum vulgare* [18], and various phosphorylation sites have already been identified in the L80 region of *Zea mays* PHO1 including threonine 537, serine 547, serine 556, serine 565, and serine 566 [85]. Interestingly, serine 69 residue of *Zea mays* PHO1 was also reported as a modifiable phosphorylation site. However, the exact fate of these phosphorylation sites has not been characterized yet.

### 6.2. Protein–Protein Interaction and PHO1

PHO1 has been reported to be involved in phosphorylation-dependent complex formation in *Zea mays*, *Oryza sativa*, *Hordeum vulgare*, and *Triticum aestivum* (Table 2).

Phosphorylation-dependent protein–protein interaction occurred in the amyloplast stroma among or between SBEI and SBEII of wheat plants harvested at mid-to-late stages of endosperm development [46]. The same interaction was not detected in early developmental stages, possibly because of the lower expression of SBEI [66]. In *Zea mays*, a novel complex was reported, which comprises large and small subunits of AGPase, SBEIIa, SBEIIb, SSIIa, SSIII, and pyruvate phosphate dikinase (PPDK) with PHO1 [91]. In *ae*^−1^ of maize, in which the amyloplast lacks SBEIIb, a novel protein–protein interaction complex was reported among or with SSI, SSIIa, SBEIa, and SBEIIa [19,51]. In *ae*^−2^, the inactive form of SBEIIb was found to be involved in complex formation with SSI, SSIIa, and SBEI both in the granule and stroma. Interestingly, PHO1 is not involved in complex formation in *ae*^−2^ as seen in the ae^−1^ [14,19]. No direct evidence has been recognized to date to illustrate the PHO1 and SBEI/SBEIIb interactions. Co-immunoprecipitation against rice PHO1 and SBEIIa antibodies has shown a weak interaction between PHO1 and SBEIIa [11,53]. The interaction of PHO1 and SBEs in rice could be indirect, as the indirect physical assembly helps to synthesize the branched glucans or maltodextrin [52]. Experiments have also been suggested a functionally active complex formation between SSIV and PHO1 [5]. Instead of SSs and SBEs, a prominent and functionally active novel heteromeric complex between PHO1 and DPE1 has been identified and stated to synthesize glucans cooperatively when developing rice seed extract was incubated with immobilized PHO1 or PHO1 without the L80 (PHO1∆L80) region from rice [10]. Interestingly it has been claimed that this interaction is independent of phosphorylation as PHO1∆L80 (no phosphorylated peptide) can also interact with DPE1, which was reportedly still active and functional. Co-immunoprecipitation and mass spectrometry experiments on PHO1 and starch synthesizing enzymes in *Solanum tuberosum* have identified DPE1 as a potential interacting protein of PHO1. These (PHO1 and DPE1) might form a metabolon and contribute to amylopectin biosynthesis [92].

All these protein–protein interactions may serve as a regulatory network that is essential for the appropriate function or regulation of enzymes. Repression or loss of function of a single protein in a complex displays up- or down-regulation of other interacted proteins and affects the functional behavior of the complex in parallel [35,93,94,95]. Together with *Arabidopsis thaliana* [96,97], *Oryza sativa* [50,98], *Zea mays* [19], *Hordeum vulgare* [99], and *Triticum aestivum* [46], several mutants lacking in starch biosynthetic enzymes have been identified that cause starch accumulation with altered structure and properties. Thus, the integral role of PHO1 in protein–protein interaction-mediated regulation of various enzymes activity needs further clarifications.

### 6.3. Redox Regulation and PHO1

Starch biosynthetic enzymes were found to be sensitive to the redox status of the cell [85,100,101,102]. A study on the AGPase enzyme indicated that both the cytosolic and plastidial forms of AGPase are up-regulated under the reductive status of the cell according to metabolic needs [100,103], suggesting the importance of redox status to control starch metabolism. A proteomic study on PHO1 of *Oryza sativa* [104] and *Triticum aestivum* [105] proposed PHO1 as a potential thioredoxin protein. There are 17 differentially expressed proteins for starch metabolism, and among these 16 proteins are reported as potential thioredoxin targets, and PHO1 is reported as one of those 16 proteins [12]. These findings suggest the redox status of the cell can control the starch metabolic enzymes including PHO1. Recently, it has been reported that the PHO1 activity is not affected by the reduced or oxidized dithiothreitol (DTT) and *Hordeum vulgare* thioredoxin system in *Hordeum vulgare*, suggesting that PHO1 is not a redox-sensitive protein [18]. The activity remains normal under redox conditions, and this allows PHO1 to improve the source/sink metabolite balance through possible interaction with PSI [12]. It is reported that PHO1 contains at least one intermolecular and multiple intramolecular disulfide bridges that could be affected under the redox environment. Furthermore, various structural changes in the PHO1 dimer have also been observed in response to the redox environment. Moreover, it was stated that the interaction between PHO1 and SBEs is dependent on the multimeric state of PHO1 [5], and this formation might also depend on redox status.

### 6.4. Transcription Factors (TFs) and PHO1

Starch synthesis and starch-associated proteins have been intensively investigated, as these two components limit the value of grain in cereal crops, but transcription factors to regulate the starch synthetic enzymes remain mainly unknown. A limited set of transcription factors have been identified to date [106] that contribute to the regulation of storage starch synthesis mainly in *Oryza sativa* [107,108], *Triticum aestivum* [109], and *Zea mays* [110,111,112]. NAM, ATAF1/2, and CUC2 (NAC) are the major families of plant-specific transcription factors (TFs) [111,113,114], which are reportedly involved directly (physical binding) or indirectly (functional interaction attributed to other binding partners) in the regulation of starch biosynthesis through modulating the starch biosynthetic enzymes [115]. Based on previously reported work on *Zea mays*, ZmEREB156 was found to actively modulate the SSIII [10,107,116]. ZmbZIP91 is similar to AtVIP1 and complements the changed phenotype of storage starch in the vip1 mutant of *Arabidopsis thaliana*. Moreover, ZmZIP91 was found in direct association with the promoters of various starch-synthesis-related genes including AGPS1, SSI, SSIIIa, and ISA1. OsbZIP33 interacts with the ACGT element in the promoter region of SBEI and *Wx* (promoter), which suggests its role in the regulation of starch synthesis [117]. Recently, it has been reported that TabZIP28 of *Triticum aestivum* and TubZIP28 of *Triticum urartu* bind to the promoter region of cytosolic AGPase to increase its activity [118]. Apart from direct binding and stated roles, ZMNAC34, ZmaNAC36, ZmMADS1a, RSR1, TaPDIL1-2, and DEP1/qPE9-1 have been identified as indirect regulators in starch biosynthesis regulation [107,110,119,120,121]. No direct or indirect interacting TFs have been found or described to date that can influence the phosphorylase. This is a critical gap in the study of phosphorylase regulation. Recently, OsNAC20 and OsNAC26 have been reported to influence the expression, RNA level, and activity of DPE1 indirectly in *Oryza sativa* [111]. This might be because of the other partner of DPE1 from starch biosynthetic enzymes. PHO1 is already reported as the functional partner for DPE1 [17] suggesting the need for a detailed study that to determine whether these transcription factors influence the PHO1 or not. The transcription factors that are reported to date mostly concern the enzymes that can form the protein–protein complex with PHO1. TFs that are stated to influence the SSs and SBEs directly could influence the expression, transcript level, or activity of PHO1 indirectly. Moreover, the presence of the L80 region in the middle and highly variable N-terminus that comprises various motifs among similar species suggests the unique regulation pattern of phosphorylase that still needs to be uncovered.

## 7. Concluding Comments and Future Prospects

In plant cells, PHO1 and PHO2 are precisely localized in amyloplasts and cytoplasm, respectively. Both isoforms share a similar mechanism of enzyme activity. The physiological functions of gPHO, maltodextrin phosphorylase, and PHO2 of plant starch phosphorylase were found to be quite similar in phosphorolytic degradation of the storage sugar moiety. However, PHO1 is a major active form of phosphorylase in plants and functions in both synthetic and degradative directions, but its role varies among different species. The possible reason for differential behavior is the presence of a unique L80 domain and the variable N-terminus sequence. The N-terminus of the protein was found to consist of targeting peptides, signal recognition peptides, and possible phosphorylation sites, suggesting a unique regulation pattern in the plants. The L80 domain was reported to be derived from an intron [122]. Even though the L80 domain can undergo proteolytic cleavage by proteasomes in *Solanum tuberosum*, the activity of PHO1 still remains intact, suggesting the irrelevance of the L80 domain in the function depiction. A possible reason for L80 domain insertion in the PHO1 could be to restrict the activity of the enzyme to starch only. Recent technologies of enzyme assays and native affinity zymograms can be used to test this hypothesis.

The L80 domain was found to consist of the PEST motif, and comparative analysis has suggested that the PEST motif is missing in PHO1 of some crops. Proteolytic cleavage is conditional and depends upon the availability of the PEST motif and/or environmental conditions. So far, studies have not been reported in cereal crops in which PHO1 can undergo proteolytic degradation. Ample work is required to determine the factors that help the PHO1 protein to remain intact and which mechanisms have been adopted to avoid cleavage. The L80 domain is found to be rich in phosphorylation sites, and it has been reported that phosphorylation of L80 at a single serine residue increases the susceptibility of the PHO1 to proteolytic degradation [68]. Determining the effect of phosphorylation on the degradation of PHO1 would be an important aim of PHO molecular studies. PEST motif and phosphorylation sites signify a definite role in regulation. Various amino acid residues of PHO1 have been identified precisely in *Zea mays*, which can undergo phosphorylation. However, the fate of these modifications has not been completely characterized yet. It has been reported that PHO1 activity can be regulated by phosphorylation [66]. A study on phosphorylation sites could be critical in the functional depiction and regulation of the PHO1 gene. Amendments (site-directed mutations) in these sites could help in the functional characterization and understanding the regulation of the PHO1 because phosphorylation is reported as the essential part of post-transcriptional modifications of the enzyme.

In relation to starch metabolism, we cannot understate the importance of PHO1. It was determined to be an integral part of starch metabolism primarily in non-photosynthetic cells, maybe because of regulating the activity of various enzymes through protein–protein interactions. Most of the isoforms of DBEs, SSs, and DPEs are reported to form complexes with PHO1; however, the exact fate of complex formation is still unclear. The classical view of starch degradation is based on the non-regulatory degradation of starch. However, recent findings have suggested critical starch degrading enzymes (amylase, GWD, PWD) that make PHO1 an indirect-acting or regulatory candidate that can influence the activity of another participating enzyme. Although PHO was discovered over 75 years ago, its precise role is still in debate. Based on biochemical evidence we can suggest that the generation of various mutant lines (lacking in PHO1) and metabolite comparison among mutants may help to elucidate its main role in the starch biosynthetic pathway.

Phosphorylases have broad potential in crop improvement due to their versatile function and structure. Various thermotolerant variants of AGPase showed thermostability in maize cultivars during heat stress. This suggests that other variants of starch enzymes can be also tested for their potential to breed high-yielding cultivars during heat stress. A few phosphorylases have already been exploited in biotechnology and industrial research due to their glycosyltransferase activity and thermostability. PHO gene can be genetically manipulated to increase the calorific value of silage feed from legumes, grasses, and cereal crops. Storage reservoirs of starch in the form of seeds can also be genetically modified to achieve early emergence and in obtaining the optimum period of seed dormancy. The quality improvements of starch phosphorylases in many crops, like wheat, potato, rice, and maize will contribute greatly to food security.

## Figures and Tables

**Table 2:** Complexes of PHO1 with various starch synthesizing enzymes in the endosperm of cereal crops.

Crop	Approximated Complex Size (kDa)	Enzymes Involved	References
Maize	300	PHO1 ^P^, SBEll, SBEllb ^P^, SSlla ^P^	[19]
Rice	230	PHO1 ^P^, SBEl, SBEllb ^P^	[50]
Barley	Not determined	PHO1 ^P^, SBEl, SBEla, SBEllb ^P^	[90]
Wheat	260	PHO1 ^P^, SSlla, SBElla ^P^, SBEllb ^P^	[46]
Maize *ae*^−^	300	PHO1 ^P^, SBEl, SBElla ^P^, SSl, SSlla ^P^	[19]

Note: *ae*^−^; amylose extender, ^P^; Phosphorylated, SBEIa; subdivision of SBEI with slightly differing catalytic properties, SSIIa; subdivision of SSII with slightly differing catalytic properties.
